# Giant Squamous Cell Papilloma of the Eyelid—Diagnostic and Therapeutic Challenges

**DOI:** 10.1155/2019/5830493

**Published:** 2019-10-29

**Authors:** Attila Vass, Gábor Vass, Erika Gabriella Kis, Levente Kuthi, Judit Oláh, Tibor Hortobágyi, Edit Tóth-Molnár

**Affiliations:** ^1^Department of Ophthalmology, University of Szeged, Szeged, Hungary; ^2^Department of Otolaryngology and Head and Neck Surgery, University of Szeged, Szeged, Hungary; ^3^Department of Dermatology and Allergology, University of Szeged, Szeged, Hungary; ^4^Department of Pathology, University of Szeged, Szeged, Hungary; ^5^Department of Oncology, University of Szeged, Szeged, Hungary

## Abstract

Squamous cell papilloma (SCP) is generally a human papillomavirus (HPV) induced exophytic or endophytic proliferation on the surface of the skin, oral cavity, larynx, esophagus, cervix, vagina, and anal canal. The endophytic type SCP can cause differential diagnostic difficulties with keratoacanthoma, inverted follicular keratosis, and squamous cell carcinoma; however, these lesions are not associated with HPV infection. The authors present a female patient who noticed an extremely rapidly growing tumor destructing the left lower eyelid. The histological analysis of the biopsy sample revealed a virus-induced squamoproliferative lesion. The eyelid affected was completely removed, and the histological examination resulted in a HPV induced endophytic squamous cell papilloma. The tarsus and the conjunctiva were replaced by a chondromucosal graft harvested from the nasal septum, while the skin defect could be closed directly. Restoration of the eyelid function has been achieved with satisfying functional and cosmetic results.

## 1. Introduction

Squamous cell papilloma (SCP) is defined as a benign proliferation of the surface epithelium of various organs including the skin, lip, tongue, oral cavity, larynx, pharynx, esophagus, cervix, vagina, and anal canal [1]. Histologically, SCPs have characteristic exophytic growth pattern, although endophytic component might be observed in some cases [2]. Considering the etiological factors, the main reason of SCP is the infection with human papillomavirus (HPV) that currently has more than 100 genotypes, but SCP is usually caused by HPV types 1, 2, 4, 7, and 26–29 [3]. The presence of HPV can be detected by molecular tests like FISH and PCR, but in the daily surgical pathology practice, the p16 immunohistochemistry is used as the gold standard marker of a transcriptionally active HPV infection [4]. When it comes to the differential diagnosis, keratoacanthoma, inverted follicular keratosis, and squamous cell carcinoma must be differentiated from endophytic type of SCP. The former two lesions are benign with prominent endophytic pattern, while the latter is a malignant tumor with cytological atypia and dermal invasion, however none of these are associated with HPV infection [5]. Here we report a case of an endophytic type of SCP of the lower eyelid treated with excision and surgical reconstruction using nasoseptal cartilage-derived mucoperichondrial flap.

The report adhered to the tenets of the Declaration of Helsinki as amended in 2013. The patient gave her informed consent prior to surgical intervention. The patient provided written consent for publication of personal identifying information including medical record details and photographs.

## 2. Case Presentation

**Figure 1:**
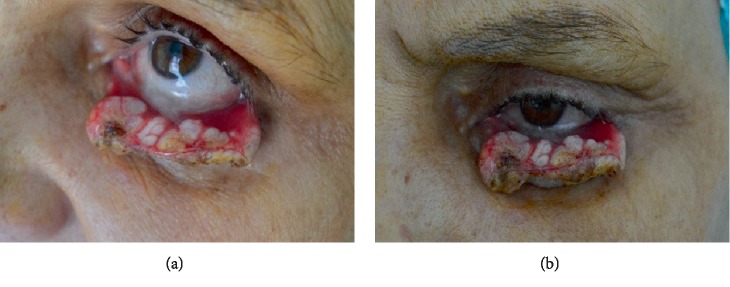
Giant squamous cell papilloma involving the left lower eyelid (a) and (b).

**Figure 2:**
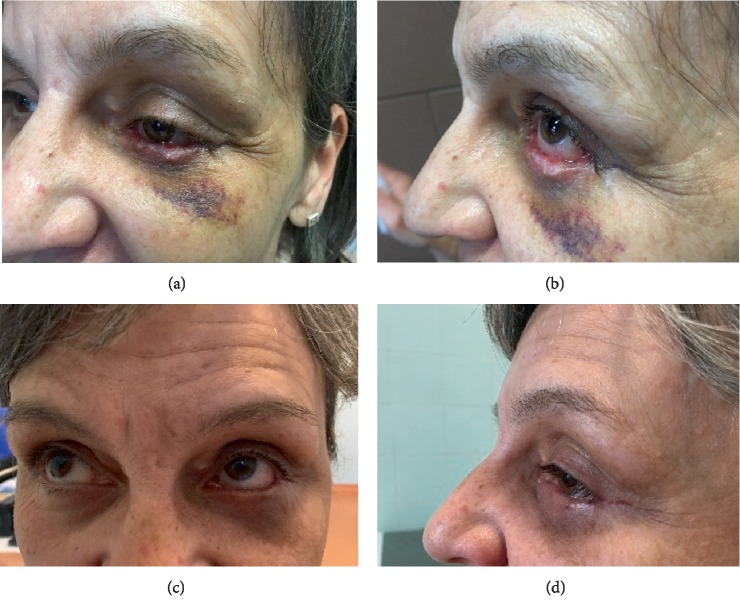
Clinical appearance of the left lower eyelid 1 week (a) and (b) and 2 months (c) and (d) after tumor removal and surgical reconstruction of the excised area.

**Figure 3:**
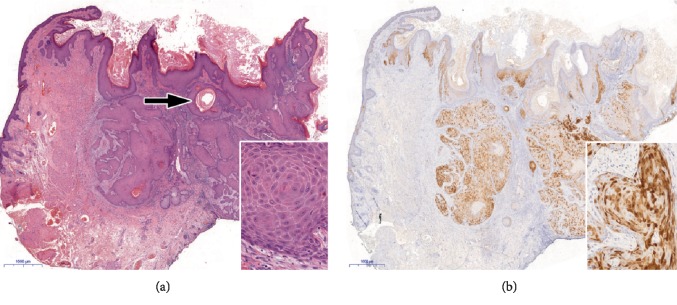
Histopathological features. (a) Hyperkeratotic epidermis and endophytic epithelial growth into the dermis with squamous pearl (arrow). There is only mild cytological atypia with intact basement membrane and no increased mitotic activity indicative of the benign nature of the lesion (inset). (b) Diffuse p16 immunopositivity both in the nucleus and in the cytoplasm (see inset) confirms HPV infection. Note the immunonegativity in the adjacent epidermis. (a: Haematoxylin & Eosin, original magnification: x20; inset: x300; b: Immunohistochemistry with DAB chromogen, using monoclonal antibody (clone MX007, Master Diagnostica; x20; inset: x300).

A 54-year-old woman without any concomitant disease was referred to our clinic with a non-pigmented tumor of the left lower eyelid measuring 21 × 11 × 19 mm, respectively. Parts of the lesion exhibited necrosis and keratinization (Figure 1). The patient had noticed a tiny, hordeolum-like lesion 10 weeks earlier, which did not respond to antibiotic ointment. Therefore, the patient was referred to our department. After detailed ophthalmological examination, biopsy was performed. The histological examination revealed a virus-induced squamoproliferative lesion. Considering the large extent of the tumor and the substantial destruction of the lower eyelid, surgical excision and oculoplastic reconstruction was performed. The excision required the removal of the entire lower eyelid except for the medial canthal area. The tarsus and the conjunctiva were replaced with a chondromucosal graft harvested from the nasal septum, while the skin was widely undermined allowing primary closure of the skin defect. New lateral canthus was formed by fixating the temporal part of the reconstructed eyelid to the periosteum of the orbital rim using deep periosteal sutures. The medial canthal area with the lacrimal punctum could be spared, allowing direct suturing of the septal cartilage (Figure 2). The histopathological examination established the diagnosis of HPV induced endophytic SCP and confirmed the complete excision of the lesion (Figure 3). The postoperative period was uneventful and the patient could be emitted 3 days after the surgical intervention. No ectropion or drooping of the lower eyelid has developed in the past 6 months, and the graft remained viable without any sign of rejection. Good functional and cosmetic results have been achieved and the patient could return to her usual life and daily activities.

## 3. Discussion

Endophytic SCP is a form of HPV induced squamous proliferation that can reach considerable size during a relatively short time interval. In our case, more than 90% of the lower eyelid was replaced by the giant tumor, causing malfunction with high risk of serious ocular surface damage. Diagnosis of endophytic SCP can pose difficulties as it can be confused with keratoacanthoma, inverted follicular keratosis, or squamous cell carcinoma based on the similarities in the clinical appearance and histopathological features. In some cases, however, it is impossible to distinguish between keratoacanthoma and squamous cell proliferations. Furthermore, biopsy diagnosis can be misleading, because typical histopathological features may only be seen in completely removed lesion [6]. In our case, the biopsy sampling revealed a virus-induced squamoproliferative lesion. Although histological analysis of the completely resected lesion raised challenging differential diagnostic questions, the diagnosis of the biopsy sampling could be confirmed. Koilocytes were seen in the epidermis and the lesion showed a “block” positivity with p16 (a protein coded by *CDKN2A* gene) immunohistochemistry, although the anti-HPV16 L1 antibody revealed negative result. The p16-positivity excluded keratoacanthoma, inverted follicular keratosis, or squamous cell carcinoma. Although various therapeutic options can be considered for large squamous eyelid tumors, such as surgical excision, topical chemotherapy, intralesional interferon, or ablative laser treatment, surgical excision remains the treatment of choice in most of the cases [7–10]. Restoration of the eyelid after excision of locally advanced lesions is challenging as functional and cosmetic results must be taken into account. Eyelids play an essential role in protection of the ocular surface and in the maintenance of its integrity. Different surgical options can be used for the replacement of the tarsus. It can be reconstructed with the mucoperiosteum of the hard palate, with auricular cartilage, with nasoseptal mucocartilage graft, or with autologous free tarsal graft [11–13]. Autologous free tarsal graft harvested from the upper eyelid can be used successfully to restore the inner lamella (both the tarsus and the tarsal conjunctiva) of the eyelid, although the size of the graft has some limitations. In our case, nasal septal cartilage graft was applied successfully for the reconstruction of the inner plate of the lower eyelid resulting in good graft viability with excellent aesthetic and function. Miyamoto et al. however recommend palatal mucosa because it has both enough stiffness and flexibility [12]. The authors consider labial mucosa too week, and auricular cartilage too stiff to replace the tarsus. According to our experience, the nasoseptal cartilage is flexible enough, and the stiffness can be enhanced by carving the graft cartilage. In our case, satisfying functional and cosmetic result could be achieved, no ulceration or ectropion was detected during the past 12 months.
